# The role and synergistic mechanisms of clinical nurse specialists in cancer multidisciplinary treatment in China: a narrative review

**DOI:** 10.3389/fpsyg.2026.1746396

**Published:** 2026-02-17

**Authors:** Xueling Jiang, Tianbo Ji, Xiufang Li, Yuting Zhang, Rong Yin, Shuhang Chen, Rong He

**Affiliations:** 1 School of Nursing, Yunnan University of Chinese Medicine, Kunming, Yunnan, China; 2 The First Affiliated Hospital of Yunnan University of Chinese Medicine, Kunming, China

**Keywords:** cancer nursing, Chinese healthcare system, collaborative mechanism, multidisciplinary team, nurse practitioner, role redefinition

## Abstract

Integrating specialized nurses into the clinical implementation pathways of multidisciplinary treatment (MDT) for cancer in China holds significant importance. This paper systematically analyzes the development of cancer MDT in China, the current status of specialized nurses’ participation in cancer MDT domestically and internationally, their core roles, and the challenges they face. Based on the characteristics of China’s healthcare system, it proposes optimization strategies including institutional empowerment, capacity building, performance incentives, and standard restructuring. The aim is to provide references for promoting the deep involvement of specialized nurses in MDT, enhancing the quality of cancer patient diagnosis and treatment, and improving the continuity of nursing services, thereby offering practical evidence for refining and promoting China’s cancer MDT model.

## 1 Introduction

With the advancement of cutting-edge medical technologies and the development of novel anticancer drugs, the complexity of cancer diagnosis and treatment continues to increase. The care and treatment of patients with advanced-stage cancer necessitates the integration of multidisciplinary professional resources (Yang et al., 2021). As the core model of integrated care, multidisciplinary treatment (MDT) establishes dedicated teams and conducts regular case conferences to develop personalized, safe, and effective treatment plans for patients. It has become a critical mechanism in global cancer care (Carvalho et al., 2023). A favorable prognosis for cancer patients depends not only on the clinical expertise of the medical team but also critically on the collaborative efforts of multidisciplinary members (Ghoshal et al., 2022; Aversano et al., 2022).

Specialized nurses, as the core force in cancer nursing, play an irreplaceable role in providing physical and psychological support to patients, facilitating team communication and coordination, and participating in treatment decision-making (Kočo et al., 2022; Fehervari et al., 2021). However, influenced by traditional hierarchical medical structures, MDT teams in China remain physician-dominated. The professional value of specialized nurses remains underutilized, with their role definition, participation pathways, and collaborative mechanisms still in the exploratory phase (Gabriel et al., 2022; Berumen et al., 2025). While domestic and international studies have confirmed the positive effects of specialized nurse involvement in MDTs, research on standardized practice pathways and mechanisms within the Chinese healthcare system remains scarce (Winters et al., 2021).

Therefore, this study focuses on the current development of cancer MDTs in China. It systematically reviews the role functions and practical challenges of specialized nurses in MDTs, explores feasible pathways for role reconstruction and strategies for optimizing collaborative mechanisms, and provides theoretical and practical support for promoting the deep integration of specialized nurses into MDTs and enhancing the overall quality of cancer diagnosis and treatment in China.

## 2 Development overview of multidisciplinary cancer care

The multidisciplinary treatment model originated in the United States during the 1960s (Berumen et al., 2025). Subsequently, it was adopted and promoted in countries such as the United Kingdom, Italy, and Germany, gradually becoming the gold standard for cancer treatment in Western developed nations (Winters et al., 2021). Internationally, MDT has established a comprehensive regulatory framework as a mandatory component of cancer care. Peer review systems ensure standardized team operations, with specialized nurses explicitly designated as core members (Taylor et al., 2013).

China accelerated MDT development starting in 2010. With the advancement of integrated healthcare service systems, over 80% of tertiary hospitals had established cancer MDT teams by 2024 (Table 1). Chinese MDTs primarily fall into three categories: outpatient MDTs, inpatient MDTs, and remote MDTs, each tailored to different stages of cancer care—screening and diagnosis, inpatient treatment, and rehabilitation follow-up, respectively (Mao et al., 2022). During the COVID-19 pandemic, remote MDTs demonstrated significant advantages in case discussion efficiency and participation rates due to their flexibility, cost control, and ability to overcome geographical limitations. A study involving 423 MDT members revealed that remote meetings significantly improved case discussions, attendance rates, and patient representation. This indicates that remote meetings can overcome temporal and geographical barriers while delivering decision-making quality comparable to in-person meetings. However, related IT infrastructure is also recognized as an urgent issue requiring resolution for the development of remote MDTs (Soukup et al., 2023).

**Table 1:** Development history and types of cancer MDT models in China.

Category	Content	Features	Applicable scenarios
Development history	1960s	Origins of MDT (Western)	Laying the foundation for multidisciplinary collaboration in cancer treatment
	2010	Launch of MDT development in china	Officially launching the localization development process promoting the standardized
	After 2020	Comprehensive policy implementation (national health commission directive)	Implementation of MDT across national medical institutions
	Present	Coverage rate in grade a tertiary hospitals >80%	Become one of the core models for cancer diagnosis and treatment
Main types	Outpatient MDT	Fixed regular outpatient hours; full participation of patients and families throughout the process	Initial case evaluation and consultation for complex cases
	Inpatient MDT	Real-time collaboration across multiple hospital departments; development of personalized treatment plans	Hospitalization for patients with advanced-stage cancer and acute conditions
	Telemedicine MDT	Cross-regional expert collaboration; supported by robust IT infrastructure	Diagnosis and treatment for patients in remote areas and rare cancer cases; overcoming geographical and time constraints

Despite China’s rapid MDT advancement, room for improvement remains in team collaboration efficiency, member role definition, and standardized processes. As a critical nursing force, the participation and contribution of specialty nurses within MDTs require further optimization.

## 3 Current status of specialist nurses in cancer multidisciplinary team care

### 3.1 International practices of specialist nurses in MDT

In Western developed countries, the role of specialist nurses first emerged in the 1970s when attention was first drawn to the psychosocial issues of breast cancer patients (Yao et al., 2023). Since then, the role of specialized nurses has continuously evolved. Currently, specialized nurses are explicitly designated as core members of cancer MDTs, with their functional roles and participation pathways forming a mature standardized system (Taylor et al., 2013; Erica et al., 2022). The UK National Health Service (NHS) mandates that urological cancer MDTs be led by specialized nurses; compared to other cancer care networks, this model improved patient management quality scores by 27% (McGlynn et al., 2017). An Italian cancer center fully integrated specialized nurses into MDT implementation pathways by establishing specialized nurse clinics. Within 1 year, MDT meeting participation rates reached 95%, with patient satisfaction exceeding 83.2% (Zeneli et al., 2021).

Internationally, specialized nurses possess high autonomy within MDTs, not only fulfilling foundational duties like patient care, education, and support but also playing leading roles in clinical decision-making and team coordination. A systematic review by Cook et al. (2021) demonstrated that deep involvement of specialized nurses significantly improves patient quality of life, nursing satisfaction, and treatment continuity. MDT services led by specialized nurses effectively bridge gaps in traditional treatment models, forming an efficient and collaborative healthcare system (Sharpley et al., 2021).

### 3.2 Current status of specialist nurses’ participation in MDT in China

The involvement of specialist nurses in cancer MDT remains in its early stages in China. However, with the growing demand for specialized cancer care, the training and deployment of specialist nurses have seen rapid development. Although not yet formally incorporated as core members of MDT teams, the role of specialist nurses in cancer diagnosis and treatment is gradually gaining recognition. A 2014 study by Yan et al. (2014) on specialized nurses participating in multidisciplinary cancer consultations revealed that 96% of physicians and 78% of patients viewed their involvement as beneficial Blaschke et al. (2019) conducted qualitative research to clarify the multifaceted roles of breast care nurses within MDTs, including data compilers, coordinators and communicators, patient advocates, and outcome implementers.

In recent years, the professional value of specialized nurses in MDTs has become increasingly prominent. They not only provide specialized consultation and guidance to patients, improve their physical and mental well-being and healthcare experience, but also ensure continuity of nursing services (Chen et al., 2021; Wang et al., 2025). MDT collaborative care models led by specialized nurses have also gradually emerged (Soukup et al., 2021; Ma et al., 2022). A randomized controlled trial by Chen et al. (2022) demonstrated that individualized care grounded in MDT principles reduced anxiety scores by 40% in breast cancer patients, significantly promoting limb function recovery and quality of life improvement.

However, the involvement of Chinese specialty nurses in MDTs still faces challenges such as regional development disparities, limited participation levels, and ambiguous role definitions. Compared to mature models in Western developed countries, significant gaps remain (see Table 2).

**Table 2:** Comparison of the current status of specialist nurses in MDT teams internationally and in China.

Comparative Dimensions	The international situation	The current state of China
Role definition	Designated as core members with decision-making authority and leadership responsibility.	Often occupying a supporting role with limited decision-making authority.
Participation pathways	Institutionalised participation, such as specialist nurse clinics and leading multidisciplinary team meetings.	Participation takes diverse forms but remains unstandardised, with coordination and education as the primary focus.
Training framework	Systematic advanced nurse practitioner training, interdisciplinary courses and certification.	Training is fragmented, lacking unified certification and interdisciplinary curricula.
Policy support	National health systems explicitly incorporate guidelines, such as those stipulated by the NHS.	Policy advocacy lags behind implementation, with no mandatory inclusion mechanism in place.
Digital infrastructure support	A mature information system supports remote participation and electronic medical record sharing.	The level of informatisation varies across regions, and remote MDT is still in the process of being rolled out.

## 4 The role function and collaborative value of Chinese specialist nurses in Cancer MDTs

Specialist nurses fulfill multiple roles within cancer multidisciplinary teams (MDTs). They serve as bridges connecting the medical team with patients while contributing unique professional value to collaborative diagnosis and treatment. Their core role functions can be summarized across four dimensions (Table 3).

**Table 3:** Core roles and collaborative value of Chinese specialty nurses in cancer multidisciplinary teams (MDTs).

Role positioning	Core functions	Collaborative value	Practical case studies
Coordinator	1. Comprehensive coordination and navigation throughout patient care; 2. Information exchange among team members; 3. Facilitating communication between medical staff and patients; 4. Integration and optimization of diagnostic and treatment processes.	1. Ensure continuity of care services; 2. Enhance team communication efficiency; 3. Reduce gaps in treatment continuity; 4. Improve patient experience.	A survey of colorectal cancer patients revealed that 90.4% acknowledged the core role of specialized nurses in coordinating care and facilitating information exchange (Kramer et al., 2023).
Educator	1. Patient and family education on disease knowledge; 2. Treatment plan and rehabilitation guidance; 3. Teaching of psychological adjustment methods; 4. Notification of follow-up treatment arrangements.	1. Increase patient treatment adherence; 2. Shorten treatment waiting times; 3. Lower complication rates; 4. Strengthen patient self-management capabilities.	Research on prostate cancer patients indicated that 96% expressed high satisfaction with the specialized consultation and treatment coordination services provided by specialized nurses (Hawks et al., 2022).
Manager	1. Comprehensive assessment of patient needs; 2. Monitoring of treatment tolerance and toxicity; 3. Development and implementation of rehabilitation plans; 4. Follow-up on MDT treatment plan implementation.	1. Improve the implementation rate of multidisciplinary team (MDT) treatment plans; 2. Promptly identify adverse reactions to diagnosis and treatment; 3. Ensure the delivery of personalized nursing care; 4. Enhance the quality of closed-loop diagnosis and treatment.	A randomized controlled trial of rectal cancer patients demonstrated significantly higher multidisciplinary team (MDT) treatment adherence (79%) in the nurse specialist-assisted group compared to the control group (28%) (Dinoy et al., 2021).
Supporter	1. Psychological support for patients and their families; 2. Advocacy and expression of patient preferences; 3. Identification and feedback of unspoken needs; 4. Provision of patient-related factors for clinical decision-making.	1. Alleviate patients’ psychological stress; 2. Ensure patient-centered diagnosis and treatment; 3. Optimize the scientific rigor of clinical decision-making; 4. Enhance patients’ sense of fulfillment with diagnosis and treatment.	Specialized nurses provide support that optimizes the scientific rigor and humanistic approach of clinical decision-making, enhancing patients’ sense of fulfillment and satisfaction with care (Sheridan et al., 2020).

### 4.1 Coordinators: the core integrators of the care process

Specialist nurses serve as the critical guarantors of continuity in cancer patient care. Functioning as central liaison points within the healthcare system, they undertake multidimensional coordination responsibilities. At the patient level, specialist nurses provide comprehensive care navigation, coordinate diagnostic and treatment schedules, and resolve practical issues encountered during the healthcare journey. At the team level, they build communication bridges between MDT members and between MDTs and primary care providers, ensuring timely and accurate information exchange (Kramer et al., 2023). A study on colorectal cancer patients revealed that 285 (90.4%) patients acknowledged the specialized nurses’ capabilities in coordinating care, facilitating investigations, and providing information to patients and families, establishing them as pivotal nodes in doctor-patient communication (Petrushnko et al., 2022). An ethnographic study by Bobat et al. (2020) confirmed that specialized nurses undertake complex coordination, relationship-building, and advanced communication tasks within MDTs, serving as vital connectors that ensure the team’s efficient operation.

### 4.2 Educators: the key force in patient empowerment

Specialist nurses play an irreplaceable role in patient education and counseling through their expertise and practical experience. Their educational content spans disease awareness, treatment plan interpretation, adverse reaction management, rehabilitation training guidance, and more. Their educational methods are more targeted and accessible, effectively addressing the time and approach limitations of medical specialists in patient education. In a survey regarding prostate cancer assessment and diagnosis, 90% (791 patients) received their prostate biopsy results from a specialized nurse. Among the 135 patients diagnosed with cancer, 90% stated they would choose this approach again, 70% perceived no drawbacks, and 96% expressed high satisfaction with the specialized nurse’s expertise, related counseling, and subsequent MDT meeting arrangements (Hawks et al., 2022). Research indicates that systematic education delivered by specialized nurses reduces the average time from referral to initial treatment for newly diagnosed cancer from 53 days to 41 days. This significantly enhances treatment adherence and self-management capabilities, shortens clinical waiting times, and lays the groundwork for the successful implementation of multidisciplinary team (MDT) protocols (Waters et al., 2021).

### 4.3 Managers: the primary guarantors of clinical quality

Specialist nurses provide comprehensive patient management throughout the treatment journey. Through holistic needs assessments and meticulous monitoring of treatment tolerance and toxicity, they deliver critical safeguards for multidisciplinary team (MDT) clinical quality (Sharpley et al., 2021). Compared to other healthcare providers, specialty nurses dedicate more time to patients, performing comprehensive needs assessments, monitoring treatment tolerance, and tracking rehabilitation progress. They promptly identify potential issues during care and relay them to the MDT team. A study on prostate cancer patients demonstrated that specialty nurses were present throughout all phases of active surveillance (AS), from patient evaluation and counseling to facilitating decisions, organizing diagnostic interventions, and ensuring patient needs were met (Bates et al., 2020). Typically, specialized nurses interact with other disciplines to provide continuous care. In a randomized controlled trial involving 119 rectal cancer patients, researchers divided participants into Group A (59 patients with specialized nurse support) and Group C (60 patients without specialized nurse support). Results showed that 79% of patients in Group A completed the multidisciplinary team (MDT)-prescribed management regimen, significantly higher than Group C (28%). Compliance in Group A was significantly superior to Group C (*p* < 0.001), fully demonstrating the critical value of specialized nurses in clinical management (Dinoy et al., 2021).

### 4.4 Advocates: spokespersons for patient rights

In cancer diagnosis and treatment, patient involvement in their own medical decisions is regarded as a key aspect of healthcare system success and sustainability. However, patients often face difficulties in directly participating in multidisciplinary team (MDT) meetings. As the team members with the closest contact to patients and their families, specialized nurses serve as the primary channel for advocating for patient rights and expressing their needs (Brown et al., 2018). Specialist nurses can accurately identify the psychological needs underlying patients’ non-physical symptoms (such as fear of death or treatment concerns), and incorporate key information including patient preferences, psychological state, and family support into multidisciplinary team decision-making (Cruciani et al., 2019). Providing support to cancer patients and families while advocating for patient wishes has also been identified as a key role of specialized nurses (Sheridan et al., 2020). Research indicates that the psychological support and needs feedback provided by specialized nurses enhance the scientific rigor and humanistic quality of clinical decisions, thereby improving patients’ sense of fulfillment and satisfaction with care (Maurizi et al., 2018; Farina et al., 2022).

In summary, the role of specialized nurses within multidisciplinary teams is crucial for enhancing the continuity of care experience for cancer patients. Particularly in Western countries, many nations emphasize the necessity of integrating specialized nurses into MDTs. However, research on specialized nurses’ participation in MDTs remains in its early exploratory stages in China. To date, only a limited number of studies have examined the clinical implementation pathways for specialized nurses’ involvement in MDTs from their perspective. Although results predominantly indicate positive effects, significant constraints still exist regarding specialized nurses’ participation in MDTs.

Specialist nurses, through comprehensive coordination, systematic education, holistic management and proactive support, not only enhance the continuity and seamless integration of nursing services but also systematically incorporate patients’ values, preferences and psychosocial needs into multidisciplinary team (MDT) decision-making processes. For instance, acting as ‘coordinators’ and ‘supporters’, specialist nurses convey patients’ unspoken needs and treatment preferences during MDT meetings, directly influencing the humanisation of treatment plans (Cruciani et al., 2019; Maurizi et al., 2018). This mechanism of deep engagement effectively elevates nursing continuity from the service level to the decision-integration level, thereby genuinely realising patient-centred MDT collaboration.

## 5 Challenges faced by Chinese specialist nurses in participating in cancer MDTs

Despite the significant value of specialist nurses in cancer multidisciplinary teams (MDTs), their deep involvement still faces multiple challenges within the Chinese healthcare system (Table 4). These challenges primarily center on four dimensions: institutional safeguards, communication and collaboration, resource support, and professional development.

**Table 4:** Key challenges and influencing factors for chinese specialty nurses participating in cancer multidisciplinary teams (MDTs).

Challenge dimensions	Specific manifestations
Institutional safeguards	1. Imbalanced decision-making authority: specialist nurses are rarely included in core MDT teams, with their opinions adopted at only 23% of the rate for physicians; 2. Ambiguous role definition: lack of unified standards for duty boundaries, participation scope, and collaboration methods; 3. Absence of Role Specifications: No standardized workflow established for specialist nurses in MDTs.
Communication and collaboration	1. Interdisciplinary barriers: traditional hierarchical mindsets and disciplinary silos hinder effective communication with other MDT members; 2. Prognosis communication dilemma: caught in the ethical conflict between “disclosing the truth” and “preserving hope,” lacking proactive engagement; 3. Suppressed meeting atmosphere: Only 51.7% of specialty nurses dare to challenge team members’ perspectives.
Resource support	1. Excessive workload: heavy routine nursing tasks leave insufficient time for MDT-related activities; 2. Lack of incentive mechanisms: benefits show no significant difference from regular nurses, with no specialized evaluations or promotion pathways tied to MDT participation; 3. Inadequate MDT understanding: 85.7% of nursing staff lack sufficient comprehension of MDT.
Professional development	1. Unstandardized training system: absence of unified certification bodies, inconsistent training content and assessment criteria; 2. Lack of interdisciplinary knowledge: insufficient expertise in oncology, pathology, radiology, etc., undermining professional influence; 3. Absence of professional authority: varied skill levels, leading to skepticism from patients and peers.

### 5.1 Institutional level: imbalanced decision-making authority and ambiguous role definition

The core characteristic of MDT is the equal participation of all members in decision-making discussions; however, Chinese MDT teams still exhibit a pronounced imbalance in decision-making authority (Askelin et al., 2021). Specialist nurses lag considerably behind physicians in terms of participation numbers, engagement levels, representativeness, and contribution. Surveys indicate that 52 out of 79 healthcare institutions exclude specialist nurses from core MDT teams, with their opinions adopted at a rate merely 23% that of physicians. Patient-related information receives the lowest scoring in MDT discussions, leading to the neglect of critical factors such as patient preferences and psychological state, thereby undermining the individualisation and humanistic aspects of nursing care (Brims et al., 2022). MDT meetings are predominantly led by physicians from disciplines such as oncology, histopathology, and diagnostics, with specialist nurses often relegated to peripheral roles, lacking equal rights to speak and participate in decision-making (Caraceni et al., 2022).

Concurrently, China lacks established role specifications and workflows for specialty nurses in MDTs. There are no unified standards defining their responsibilities, scope of participation, or collaboration methods within these teams. Some specialty nurses hesitate to engage in MDT meetings due to unclear role definitions, hindering their ability to fully leverage their professional expertise. A cross-sectional study analyzing 223 MDT cases across six hospitals revealed that specialized nurses contributed significantly less than other team members (Gandamihardja et al., 2019). Disease-centered information dominated MDT decision-making, while patient-centered factors (such as patient preferences and comorbidities) were secondary (Wihl et al., 2021). A case review of 349 patients revealed that radiology information scored highest, followed by histopathology, while patient-related information scored lowest (Bruun et al., 2022). Traditional hierarchical structures, core member absences, and unequal decision-making power pose disruptive risks. These factors foster bias and groupthink within MDTs, marginalizing specialized nurses and undermining the very purpose of collective decision-making. This structural inequality in decision-making authority directly constrains specialist nurses from fulfilling their role as patient advocates and humanistic care liaisons within multidisciplinary teams. Due to a lack of equal voice, critical information such as patient preferences and psychosocial needs is frequently marginalised in MDT discussions (Brims et al., 2022). This results in inadequate personalisation of treatment plans and insufficient humanistic care, thereby compromising the overall quality of patient-centred holistic care.

### 5.2 Communication level: barriers to interdisciplinary collaboration and communication challenges

The complex work environment in cancer subjects specialized nurses to dual pressures in communicating with patients and multidisciplinary teams, intensifying their ethical dilemmas and ethical decision-making capabilities, leading to emotional exhaustion among specialized nurses (Banerjee et al., 2017). Communication between oncology nurses and team members faces significant challenges due to factors such as hierarchical status (Price et al., 2014), the independent nature of disciplines and their distinct training approaches (Leonard et al., 2004), and multidisciplinary team roles (Rowlands and Callen, 2013). A survey of cancer nurses revealed the complexity of their engagement attitudes: whilst the majority (178/230) expressed willingness to actively participate in multidisciplinary team (MDT) meetings, only approximately half (92/178) felt confident enough to challenge other team members’ viewpoints during these sessions. Furthermore, 35 nurses admitted feeling intimidated by the atmosphere within MDTs (Stewart et al., 2018). This contradictory mindset, which combines a willingness to participate with a reluctance to speak up or even feelings of intimidation, concretely reflects both hierarchical communication barriers and a suppressive team atmosphere.

Furthermore, communicating with cancer patients presents difficulties, particularly regarding diagnosis and prognosis. Cancer patients often exhibit ambivalent attitudes toward diagnostic and prognostic information, oscillating between wanting to know and not wanting to know. Physicians, meanwhile, tend to convey diagnoses fully while avoiding detailed prognosis discussions to preserve patients’ hope. A phenomenological study by Melis et al. (2020) revealed that specialty nurses often perceive the disclosure of diagnosis and prognosis as a conflicting value (positive value: empowering patient autonomy; negative value: incompatibility with patient wishes). In communicating prognostic information, specialty nurses frequently face the ethical dilemma of balancing “telling the truth” with “preserving hope,” with most viewing prognosis communication as the physician’s responsibility and lacking awareness or capacity for proactive involvement.

### 5.3 Resource level: excessive workload and lack of incentive mechanisms

The current state of strained medical resources and personnel shortages in China subjects specialty nurses to heavy workloads. Even after obtaining specialty nurse certification, they must still handle substantial routine nursing tasks, leaving them with insufficient time and energy to participate in multidisciplinary team (MDT) activities. A UK survey revealed that 63.5% of specialty nurses worked over 10% overtime weekly, with over half reporting inadequate administrative support (Stewart et al., 2018). This phenomenon is even more pronounced in China.

Concurrently, professional incentive mechanisms for specialty nurses are severely deficient. In terms of benefits and compensation, there is little discernible difference between specialty nurses and general cancer nurses, failing to reflect their specialized value. Regarding career development, the absence of specialized assessments, training, and promotion pathways for MDT participation dampens their enthusiasm. Furthermore, the fluid nature of their roles within MDTs, in which they often serve multiple functions, frequently leads to role ambiguity. A lack of understanding about MDTs among 85.7% of nursing staff further undermines participation effectiveness (Khan et al., 2023). Research by Maharaj et al. (2021) indicates that most other MDT members have experienced role overlap with specialized nurses. Furthermore, specialized nurses lack confidence in voicing opinions within their own areas of expertise (Cook et al., 2019). Although they perceive themselves as patient advocates capable of providing supportive care when needed, expressing their recommendations during MDT meetings proves challenging, leading them to prefer discussing matters privately with physicians (Findlay et al., 2021).

### 5.4 Professional level lack of standardized training systems and authoritative oversight

The development of specialty nurses in China remains in its infancy, lacking unified certification bodies and training standards. Currently, specialty nurse certifications include both those issued by the Chinese Nursing Association and those recognized by provincial health departments and hospitals. Significant discrepancies exist in training systems, curricula, and assessment criteria. This unstandardized training system results in inconsistent professional competence among specialty nurses, compromising training quality and making their professional authority vulnerable to questioning by patients and peers.

Furthermore, specialized nurses often lack sufficient interdisciplinary knowledge, making it difficult for them to contribute professional insights on other disciplines during MDT meetings. This limitation also diminishes their voice and influence within the team. Some specialized nurses, lacking confidence, prefer to discuss patient issues privately with physicians rather than openly express their recommendations during MDT meetings (see Table 5).

**Table 5:** Principal issues and challenges faced by nurses in MDT meetings.

Problem categories	Specific manifestations	Impact
Communication barriers	Strong hierarchical mindset, disciplinary barriers, limited opportunities to speak.	Nurses’ opinions struggle to gain traction, with decision-making skewed towards medical dominance.
Psychological pressure	Oppressive meeting atmosphere, fear of being challenged, ethical conflicts (e.g., discussing prognosis).	Low engagement levels and emotional exhaustion.
Resource constraints	Insufficient time, lack of administrative support, inadequate IT infrastructure.	Inadequate meeting preparation and difficulties with remote participation.
Lack of professional confidence	Shortage of interdisciplinary knowledge, unsystematic training, absence of internationally recognised benchmarks.	Restricted speaking opportunities and diminished influence.
Absence of incentive mechanisms	No dedicated assessment framework, unclear career progression pathways, inadequate financial compensation.	Lack of motivation to participate and marginalised roles.

## 6 Recommendations

In response to the aforementioned challenges, and considering the characteristics of China’s healthcare system and the current state of MDT development, the following optimization strategies are proposed to advance the role redefinition of specialty nurses within cancer MDTs and enhance collaborative mechanisms.

### 6.1 Institutional empowerment clarify role positioning and decision-making authority

#### 6.1.1 Refine MDT system standards

It is recommended that health administrative departments collaborate with medical institutions to develop the “Cancer MDT Specialist Nurse Practice Standards,” formally integrating specialist nurses as core MDT members. This should clarify their responsibilities and authority in case discussions, treatment plan formulation, and process coordination, granting them equal voting rights and decision-making authority.

#### 6.1.2 Establish an independent nursing department

Create a dedicated MDT nursing management unit responsible for selecting, training, evaluating, and coordinating specialized nurses. Regularly assess the effectiveness of specialized nurses’ participation in MDTs and promptly optimize workflows.

#### 6.1.3 Strengthen patient advocacy mechanisms

Specialist nurses should comprehensively gather information on patients’ treatment preferences, psychological states, and family support. During MDT meetings, they should promote the team’s consideration of patient factors through non-confrontational methods such as information sharing, posing questions, and offering practical suggestions. This ensures the individualization and humanistic approach of treatment plans.

### 6.2 Capacity building strengthening interdisciplinary communication and collaboration skills

#### 6.2.1 Implement multidisciplinary collaborative training

Establish a cross-disciplinary training framework covering foundational knowledge in oncology, pathology, radiology and related disciplines, alongside generic competencies such as communication skills, ethical decision-making and team collaboration. Employ diverse training methods including lectures, seminars, case studies and scenario simulations to enhance specialist nurses’ interdisciplinary literacy and communication abilities. Training content design should reference the International Council of Nurses’ position statement and core competency framework for advanced practice nurses. Current specialist nurse training in China exhibits significant gaps compared to global advanced practice standards in areas such as advanced health assessment, evidence-based practice leadership, complex clinical decision-making, and prescribing authority (e.g., for symptom management medications). Bridging these gaps is crucial for enhancing specialist nurses’ professional credibility, critical thinking, and confidence in autonomous decision-making within multidisciplinary team meetings.

#### 6.2.2 Cultivate critical thinking

Integrate training on identifying groupthink and expressing dissent, encouraging specialty nurses to proactively share professional insights during MDT meetings. Foster reasonable questioning to drive optimization of treatment plans.

#### 6.2.3 Enhancing humanistic care capabilities

Strengthen training in psychological communication and ethical decision-making for specialty nurses. Equip them with techniques for conveying diagnostic and prognostic information, balancing patients’ right to informed consent with their need for psychological support, and effectively navigating ethical dilemmas in communication.

### 6.3 Resource support establishing career development and incentive mechanisms

#### 6.3.1 Optimize workload allocation

Clearly define the scope of practice between specialty nurses and general nurses, reducing routine nursing tasks for specialty nurses to allocate sufficient time for MDT meetings, case preparation, and patient follow-ups.

#### 6.3.2 Establish targeted incentive mechanisms

① Financial Incentives: Provide specialized financial subsidies to specialized nurses participating in MDTs. ② Performance-Based Incentives: Incorporate MDT participation into nursing performance evaluations, awarding bonus points to high performers. ③ Talent Support: Create an MDT specialized nurse talent fund, distributing targeted rewards based on work quality assessment outcomes.

#### 6.3.3 Improve administrative support systems

Establish an administrative accountability system to ensure transparency and implementation of incentive policies.

Strengthen IT infrastructure to provide technical support for specialized nurses participating in remote MDTs, accessing case materials, and coordinating treatment processes.

### 6.4 Standard reconstruction refine training and certification systems

#### 6.4.1 Establish a unified certification authority

Led by the national health administration, establish a unified national certification body for specialty nurses. Develop standardized certification qualifications, training systems, and assessment criteria to regulate the training and management of specialty nurses.

#### 6.4.2 Standardize selection and training processes

Define selection criteria for MDT specialty nurses, prioritizing those with cancer nursing experience, interdisciplinary knowledge, and strong communication skills. Develop personalized training plans to enhance professional competence through mentorship programs, MDT expert lectures, and interdisciplinary rotations.

#### 6.4.3 Strengthen professional authority

Encourage specialty nurses to participate in MDT-related research and academic exchanges, publishing professional papers and research findings. Establish a specialty nurse performance evaluation system, conducting comprehensive assessments across dimensions such as work performance, professional competence, teaching/training, and patient satisfaction to enhance their professional authority and industry recognition.

### 6.5 Technology-enabled enhancement: strengthening digital and IT support for MDTs

#### 6.5.1 Establishing an integrated MDT information platform

Develop or implement a collaborative MDT system integrating functions such as medical record retrieval, imaging resource sharing, meeting minutes, and task allocation. This supports specialist nurses in efficiently preparing patient documentation pre-meeting, conducting real-time recording and feedback during sessions, and following up on implementation post-meeting.

#### 6.5.2 Promote remote MDT conferencing systems

Enhance remote conferencing hardware and software infrastructure to ensure specialist nurses can participate in MDT discussions via video or audio means, even during peak clinical workloads or when geographically dispersed. This will increase their attendance rates and opportunities to contribute.

#### 6.5.3 Strengthen data security and privacy protection

While advancing digitalisation, establish a rigorous data security management system. This will ensure the transmission, storage, and use of patient information within the MDT platform complies with ethical and legal standards.

### 6.6 Cultural transformation fostering MDT cultural reform and interprofessional respect

#### 6.6.1 Implementing interprofessional team-building training

Regularly organise MDT members to participate in training on team collaboration, communication skills, and emotional management to enhance mutual understanding and trust, breaking down disciplinary barriers and hierarchical perceptions.

#### 6.6.2 Establish feedback and recognition mechanisms

Encourage specialist nurses to contribute professional insights during MDT meetings, with public acknowledgement of their contributions. Incentive schemes such as the “Best Nursing Advice Award” or “Patient Advocate Award” may be introduced to boost engagement.

#### 6.6.3 Promote a patient-centred collaborative culture

Reinforce MDT consensus on the “patient-centred” philosophy, clarifying the vital role of specialist nurses as patient advocates to facilitate their greater involvement in decision-making.

## 7 Discussion

The advancement of multidisciplinary team (MDT) care has propelled cancer diagnosis and treatment toward shared decision-making, while also creating new development opportunities in nursing. As the core force in cancer nursing, the deep involvement of specialized nurses in MDTs is a key measure to enhance clinical quality, ensure continuity of care, and embody the patient-centered philosophy. Through systematic analysis of the role functions, current practices, and challenges faced by specialized nurses in Chinese cancer MDTs, this study proposes a four-pronged optimization strategy encompassing institutional empowerment, capacity building, resource allocation, and standard restructuring. This framework provides a practical pathway for redefining the role of specialized nurses and refining collaborative mechanisms.

Within China’s healthcare system, the benefits of specialized nurses participating in MDTs have been preliminarily demonstrated, yet multiple challenges persist, including imbalanced decision-making authority, communication barriers, insufficient resources, and lack of standardized protocols. Addressing these issues requires collaborative efforts from health administrative departments, medical institutions, MDT teams, and specialized nurses themselves: Health administrative departments should leverage top-level design to establish robust institutional frameworks; medical institutions must enhance resource support and incentive mechanisms to foster an enabling environment for specialized nurse involvement in MDTs; MDT teams should foster an atmosphere of equal respect and collaboration, fully recognizing the professional value of specialty nurses; specialty nurses themselves must proactively enhance their capabilities and actively engage in MDT practices.

Firstly, the structural inequality in decision-making authority within Chinese cancer multidisciplinary teams (MDTs) marginalises patient-centred information—such as preferences and psychosocial needs—during discussions. This limits specialist nurses’ capacity to translate critical patient factors into effective care decisions. Secondly, enhanced involvement of specialist nurses, precisely through their roles as “coordinators” and “supporters”, ensures seamless continuity across treatment phases. By acting as “patient advocates” within MDTs, they systematically integrate patient preferences into treatment plans, thereby directly strengthening nursing continuity and the human-centred nature of decision-making. Moreover, the core divergence between Chinese and Western MDT models regarding nursing autonomy manifests as institutionalised role recognition versus ambiguous auxiliary positioning, and comprehensive decision-making participation versus limited coordination of implementation. China’s localised reforms should draw upon Western approaches to institutionalising and standardising nursing roles, yet must be embedded within the organisational context of its healthcare system. This requires a phased empowerment strategy, such as granting decision-making authority on nursing-related matters, coupled with interdisciplinary capacity building and information technology enablement. Gradually, this will forge a collaborative model that integrates international perspectives with distinctive Chinese characteristics.

Future research should further explore standardized workflows for specialty nurse participation in MDTs, develop role models and collaborative mechanisms tailored to China’s cultural context, validate the effectiveness and sustainability of optimization strategies through large-scale, long-term empirical studies, Simultaneously, attention should be paid to the differentiated needs of various regions and healthcare institutions of different levels to develop tiered and categorized implementation plans. Although specialized nurses currently face numerous challenges in participating in cancer MDTs, with the continuous improvement of China’s healthcare system and the ongoing development of the MDT model, the professional value of specialized nurses will gain further recognition, and their roles and contributions in cancer diagnosis and treatment will continue to expand and deepen.
